# Non-Radiographic Risk Factors Differentiating Atypical Lipomatous Tumors from Lipomas

**DOI:** 10.3389/fonc.2016.00197

**Published:** 2016-09-22

**Authors:** Justin E. Bird, Lee Jae Morse, Lei Feng, Wei-Lien Wang, Patrick P. Lin, Bryan S. Moon, Alexander J. Lazar, Robert L. Satcher, John E. Madewell, Valerae O. Lewis

**Affiliations:** ^1^Department of Orthopaedic Oncology, MD Anderson Cancer Center, Houston, TX, USA; ^2^Department of Biostatistics, MD Anderson Cancer Center, Houston, TX, USA; ^3^Department of Pathology, MD Anderson Cancer Center, Houston, TX, USA; ^4^Sarcoma Research Center, Houston, TX, USA; ^5^MD Anderson Cancer Center, Houston, TX, USA; ^6^Department of Diagnostic Radiology, MD Anderson Cancer Center, Houston, TX, USA

**Keywords:** atypical lipomatous tumor, lipoma, liposarcoma, well-differentiated liposarcoma

## Abstract

**Purpose:**

To determine non-radiographic risk factors differentiating atypical lipomatous tumors (ALTs) from lipomas.

**Methods:**

All patients with deep-seated lipomatous tumors of the extremities treated from January 2000 to October 2010 were retrospectively reviewed. Factors reviewed included age, gender, tumor location, size, histology, local recurrence, dedifferentiation, and metastasis. Multivariate logistic regression models were used to evaluate the effects of patient characteristics on ALT status.

**Results:**

Ninety-four lipomas and 46 ALTs were included. Patients with an ALT were older (median: 60.5 vs. 55 years). Lipomas were evenly distributed between upper (48.9%) and lower extremities (51.1%), whereas ALTs predominately involved the lower extremities (91.3%). Median ALT size (22 cm) was greater than lipomas (10 cm), *p* < 0.0001. One lipoma (1.04%) recurred at 77 months and five ALTs (10.9%) recurred at an average of 39 months (19–64 months). Two ALTs originally treated with wide resection recurred with a dedifferentiated component and were treated with wide re-excision and chemotherapy. No metastases or tumor-related deaths occurred in either group at the time of last follow-up. Patients older than 60 years, tumors greater than 10 cm, or thigh location, were more likely to be diagnosed with an ALT (*p* < 0.05).

**Conclusion:**

Lipomatous tumors were more likely to be ALTs when the tumor was at least 10 cm in size, located in the thigh, or found in patients that were 60 years of age or older. These risk factors may be used to guide management and surveillance strategies, when lipomatous tumors do not display characteristic radiographic features.

## Introduction

Lipomatous tumors of the extremities are the most common soft tissue tumors encountered in clinical practice with the majority of these tumors being lipomas and atypical lipomatous tumors (ALTs) (1–3). ALTs account for 46% of all liposarcomas with the other sub-types being less frequent, which include myxoid/round cell (28%), dedifferentiated (18%), and pleomorphic liposarcoma (8%) (4). Atypical lipomatous tumor (ALT) is synonymous with well-differentiated liposarcoma (5), which is a low grade tumor that is usually deep-seated and located in the buttock, thigh, and retroperitoneal areas. ALTs can be locally aggressive (6–8) but have very low potential to metastasize or dedifferentiate (5). Histologically, they are composed of variable amounts of mature adipose tissue interspersed with enlarged atypical adipocytic and stromal cells with hyperchromatic nuclei and prominent, thickened fibrous bands. Lipomas on the other hand are benign tumors composed of mature fat that present as solitary, slow-growing, and painless masses in the subcutaneous tissue; however, they may also be deep to the fascia or intramuscular.

Characteristic radiographic features differentiate ALTs from other liposarcoma sub-types, but differentiating between a lipoma and an ALT often presents a diagnostic dilemma. Lipomas and ALTs have relatively homogenous MR signal, usually equivalent to normal fat, and generally do not enhance after gadolinium administration. Some radiographic features that suggest an ALT rather than a lipoma are larger size, thick septae, more nodular appearance, less fat content, and calcification (1, 4, 6, 7, 9–12).

Diagnostic uncertainty arises when a large homogenous lipomatous tumor does not display radiographic features consistent with an ALT. When an asymptomatic lipomatous mass exhibits features consistent with the diagnosis of an ALT, the clinician may recommend excision, whereas a lipoma may be observed. Furthermore, length and intensity of follow-up may be affected by equivocal histologic diagnoses, whereby a definitive diagnosis of ALT cannot be established.

Here, we present a large cohort of patients that underwent excision of their lipoma or ALT with the purpose of determining non-radiographic risk factors that may help to preoperatively risk stratify these two entities. These risk factors will help guide treatment for patients presenting with equivocal radiographic features.

## Materials and Methods

A retrospective chart review was performed in accordance with the MD Anderson Institutional Review Board. The orthopedic oncology database was queried to identify all patients with a histopathologic diagnosis of lipoma or ALT of the extremities treated at our institution from January 1, 2000 to October 1, 2010. Tumors were excluded if they did not have a final pathologic diagnosis of lipoma or ALT, or underwent previous excision at an outside facility. All tumors were deep to the fascia. Factors reviewed included: age, gender, location of lesion, size of lesion based on preoperative MRI (greatest single dimension measured in any direction), gross pathologic size, biopsy and final histologic diagnosis, and history of local recurrence, dedifferentiation or metastasis. Length of follow-up was determined as time from surgery to last clinic appointment, telephone follow-up, or survey. One hundred sixty-six deep-seated lipomatous tumors were marginally excised during the study period. Twenty-six patients were excluded; 10 ALTs and 4 lipomas had undergone previous excision, 6 tumors were diagnosed as lipomas but the initial histopathologic diagnosis could not exclude ALT nor could the independent blinded review, 1 ALT had myxoid features, 2 tumors had a final histopathologic diagnosis of fibrolipoma, 2 were diagnosed as hibernoma, and 1 lipoma had a hemagioma within the lesion. The size of the lesion in the greatest dimension was determined by measurements obtained from the preoperative MRI, ultrasound, or measurement of the gross specimen if no preoperative imaging was available. The location of the lesion was classified as upper extremity (arm and forearm), shoulder, or lower extremity (thigh and leg). A tumor in the hand (two lipomas) was classified as a forearm lesion, and a tumor in the ankle (one lipoma) or foot (one lipoma) was included in the leg classification.

Two musculoskeletal pathologists performed a blinded, independent, randomized review of the histologic slides to obtain diagnoses for comparison to the original, unblinded diagnoses. Preoperative radiologists’ and intraoperative surgeons’ differential diagnoses were noted if included in the preoperative radiology report or operative report, respectively. These differential diagnoses were compared with the final pathologic diagnosis.

Fisher’s exact test or Chi-square test was used to evaluate the association of age or tumor location with the diagnosis of lipoma or ALT. Wilcoxon rank sum test was used to determine differences in age and tumor size between patients with a lipoma or ALT. BLiP plots were generated for overall tumor size by lipoma and ALT diagnoses, as well as thigh location only. Multivariate logistic regression models were used to evaluate the effects of patient characteristics (age ≥60 years, gender, size > 10 cm, or thigh location) on ALT status. *p* Values less than 0.05 were considered statistically significant. Statistical software SAS 9.1.3 (SAS, Cary, NC, USA) and S-Plus 8.0 (TIBCO Software Inc., Palo Alto, CA, USA) were used for all analyses.

## Results

One hundred forty lipomatous tumors of the extremities met inclusion criteria; 94 had a final diagnosis of lipoma and 46 were ALTs. Patient demographics and tumor location are shown in Table 1. Patients with an ALT tended to be older with a median age of 60.5 years (range = 13–84) vs. 55 years (range = 17–85) for patients diagnosed with a lipoma (*p* = 0.017). The location of lipomas and ALTs differed significantly (*p* < 0.0001). Lipomas were evenly distributed between the upper and lower extremities, 48.9 and 51.1%, respectively. Whereas, ALTs predominately involved the lower extremity (91.3%), 78.3% of which were located in the thigh.

**Table 1:** **Patient demographics and tumor location**.

	Lipoma (*n* = 94)	ALT (*n* = 46)	*p* Value
Sex			0.12
Male	40 (42.6%)	26 (56.5%)	
Female	54 (57.4%)	20 (43.5%)	
Age [mean, median, years (range)]	53.3, 55 (17–85)	58.6, 60.5 (13–84)	0.017
Location			<0.0001
Lower extremity	48 (51.1%)	42 (91.3%)	
Thigh	37 (39.4%)	36 (78.3%)	
Leg	11 (11.7%)	6 (13%)	
Upper extremity	46 (48.9%)	4 (8.7%)	
Shoulder	18 (19.1%)	0 (0%)	
Upper arm	16 (17.0%)	2 (4.3%)	
Forearm	12 (12.8%)	2 (4.3%)	

*Age was compared using the Wilcoxon rank sum test. Gender and tumor location was compared using Chi-square and Fisher’s exact test, respectively*.

Table 2 shows the mean tumor size by location. Overall, lipomas had median size of 10 cm in greatest dimension (range = 0.8–35 cm), while ALTs were significantly larger at 22 cm (range = 3.3–35 cm, *p* < 0.0001). Almost all ALTs were greater than 10 cm, 93.5 vs. 53.2% of lipomas, *p* < 0.0001. In the lower extremity, ALT size was significantly larger than lipomas; median size = 22 vs. 11.8 cm, respectively (*p* < 0.0001). ALTs tended to be larger in the upper extremity but this was not significant as there were too few ALTs available for comparison. ALTs in the thigh (22 cm) were significantly larger than lipomas (12.5 cm, *p* = 0.0001); however, there was significant overlap between the two groups (Table 2, bottom right).

**Table 2:** **Median tumor size by location (greatest dimension, cm)**.

	Lipoma (*n* = 94)	ALT (*n* = 46)	*p* Value
All locations	10 (0.8–35)	22 (3.3–35)	<0.0001
Lower extremity	11.8 (0.8–35)	22 (9–35)	<0.0001
Thigh	12.5 (3–35)	22 (9–35)	0.0001
Leg	5 (0.8–24)	22 (18–27)	0.0057
Upper extremity	8.8 (1.8–19)	10.2 (3.3–26)	0.77
Arm	9.5 (3.5–15.5)	9.3 (3.3–15.3)	0.94
Forearm	7.5 (1.8–19)	15.5 (5–26)	0.58
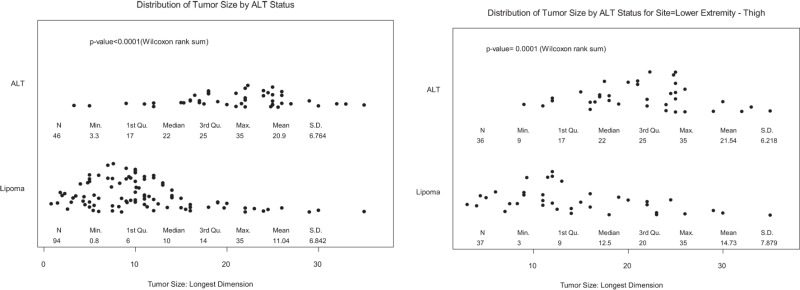			

*Median size difference between lipomas and ALTs were compared using the Wilcoxon rank sum test. Ranges are in parentheses. BliP plots are shown comparing size distribution of all ALTs and lipomas (lower left) and ALTs and lipomas in the thigh (lower right)*.

Blinded, histological review confirmed the diagnosis of ALT in all cases of previously diagnosed ALT. On review of six cases initially diagnosed as lipoma, the pathologists could not entirely exclude ALT on purely histopathologic grounds and therefore these cases were excluded from analysis. None of these six cases recurred.

The average follow-up for lipomas was 35.4 months (0–139 months) and 61.3 months (1–166 months) for ALTs. Of the 140 tumors excised, there was 1 (1.04%) local recurrence in the lipoma group and 5 (10.9%) recurrences in the ALT group (Table 3). Recurrence of the lipoma occurred 77 months after the index procedure in the ipsilateral arm but the patient chose to not re-excise the lesion. The average time to recurrence for the five patients with ALTs was 39 months from excision (19–64 months). Two of these patients chose to observe only, one patient had two subsequent recurrences after re-excisions, one patient underwent re-excision and is without recurrence 10 months after his last surgery, and one patient died 60 months after his last surgery with no reported recurrence.

**Table 3:** **Recurrent lipoma and ALT characteristics**.

	Age (years)	Sex	Site	Size (cm)	Time to recurrence (months)	Outcome
Lipoma						
Patient 1	50	F	Forearm	6	77	Treated with observation only
ALT						
Patient 1	61	F	Leg	21.6	43	Two recurrences treated with re-excisions
Patient 2	74	M	Thigh	18	64	Re-excised. No recurrence at 5 years. Unrelated death 10 years following 1° surgery
Patient 3	58	F	Thigh	17	19	Treated with observation only
Patient 4	49	M	Leg	27	33	Treated with observation only
Patient 5	64	F	Thigh	35	36	Re-excised. No recurrence at 10 months

*Time to recurrence calculated as time from primary excision until detection of recurrence*.

**Figure 1:**
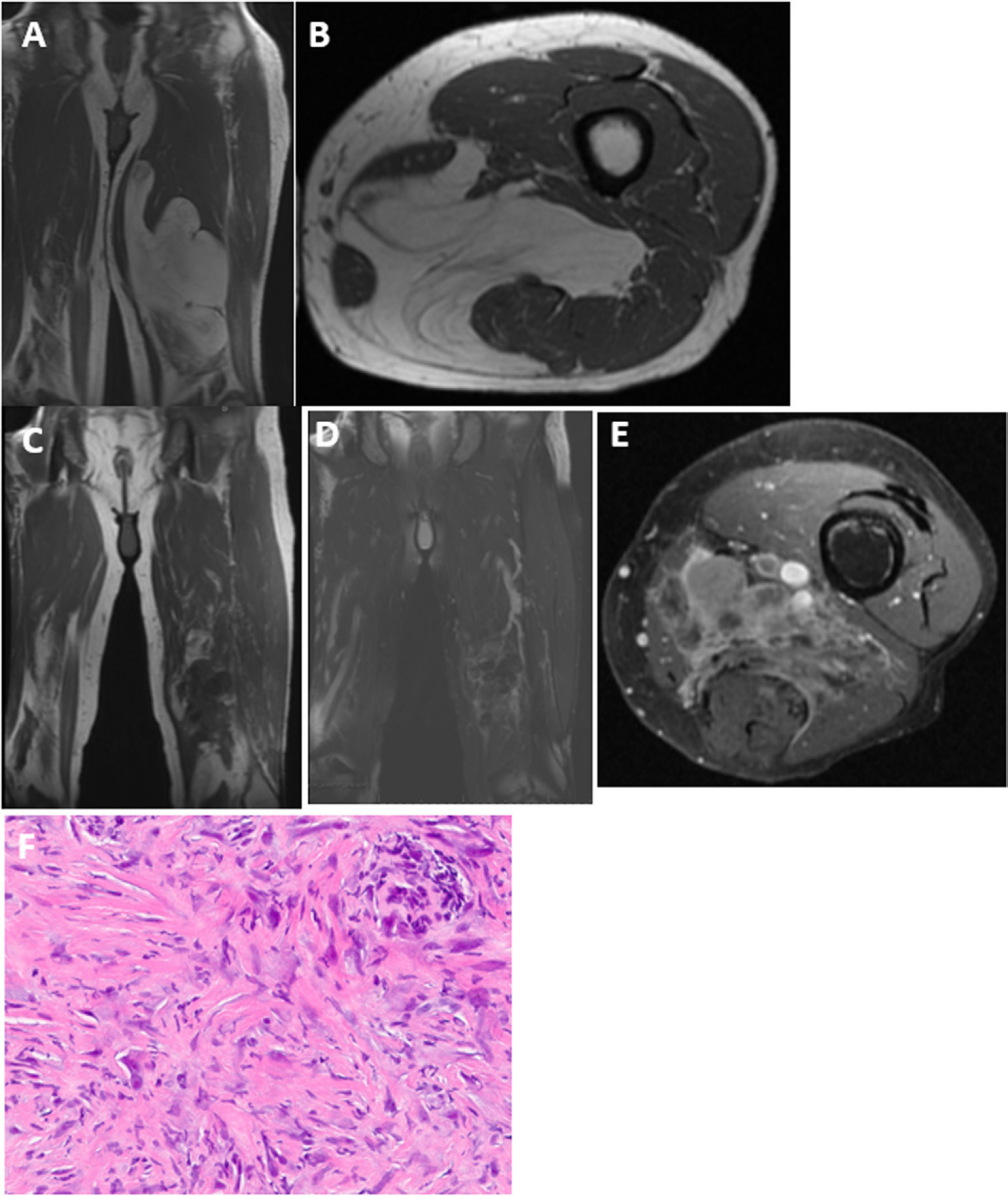
**Upon initial presentation, (A) coronal and (B) axial T1 MRI showed a large deep-seated lipomatous mass within the medial thigh**. The majority of the mass is lipomatous with some septations consistent with an ALT, which was confirmed on histology. Seven years following resection, MRI now shows a heterogenous lesion within the same region with very little fat signal that is dark on T1 **(C)** with intermediate signal on T2 **(D)** and enhances with gadolinium contrast **(E)**. **(F)** Needle biopsy shows scattered pleomorphic spindle cells confirming the diagnosis of dedifferentiated liposarcoma.

Two patients re-presented with dedifferentiated liposarcoma in the location of the previously excised ALT. One patient was a 68-year-old man who underwent marginal resection of a left medial thigh ALT. He was followed with repeat MR imaging for the next 3 years without recurrence but developed thigh swelling 7 years following his surgery. His imaging showed an enhancing heterogenous mass in the distal medial thigh at the same location of his prior resection (Figure 1). Needle biopsy was consistent with dedifferentiated liposarcoma. The second patient was a 66-year-old man who initially underwent a marginal resection of an upper arm ALT. He re-presented 42 months following his surgery with a recurrent mass that was consistent with dedifferentiated liposarcoma.

A multivariate logistic regression model was constructed to evaluate age, gender, tumor size, and thigh location as risk factors for having a diagnosis of ALT (Table 4). The median age for the ALT group was 60.5, and the median age for the Lipoma group was 55. We used the median age from the ALT group as the cutoff point since this was the cutoff also used in other studies (11). Patients who were 60 years of age or older, had tumors of at least 10 cm in size, or had tumors located in the thigh were at increased risk of being diagnosed with an ALT vs. a lipoma, *p* < 0.05. Male sex trended toward increased risk of an ALT diagnosis at significance level of 0.05.

**Table 4:** **Age, gender, size, and thigh location as risk factor for ALT**.

	Odds ratio	95% CI	*p* Value
Age >60 vs. <60 years	2.67	1.14–6.25	0.024
Male vs. female	2.28	0.97–5.37	0.059
Tumor size >10 vs. <10 cm	8.11	2.20–29.92	0.0017
Thigh location vs. all other sites	4.06	1.62–10.18	0.0028

*Multivariate logistic regression model was fitted to assess the effects of important patient characteristic variables on ALT status*.

## Discussion

Radiographic characteristics, such as size, percent fat, thickness of septae, and nodularity, are features that may help distinguish ALTs from lipomas. Frequently, however, ALTs do not display these radiographic features and lipomas may also display some features suggestive of an ALT, leading to diagnostic uncertainty. Since ALTs are more apt to recur and have the potential to dedifferentiate, discerning ALTs from lipomas is clinically important.

In our cohort of 140 deep-seated lipomatous tumors, we found that age of 60 years or older, size at least of 10 cm, and a thigh location were significant risk factors for an ALT diagnosis. This is in agreement with others who have reported that larger tumor size (11, 13, 14) and older age (11, 15) are risk factors associated with a diagnosis of ALT. Kransdorf et al. (11) found that male gender was a significant risk factor for a diagnosis of ALT. Our results were similar, male gender reached marginally significance in a multivariate analysis (*p* = 0.059).

Histologic subtyping of the ALTs has been reported to provide important prognostic information regarding recurrence rates (11). Although, histologic subtyping was not being routinely performed during the time period of this retrospective review, it is, currently being more commonly performed at our institution. However, this may not be the case at many institutions. Histologic subtyping of lipomatous tumors may not even be available at some institutions, nor is it feasible to perform on every case. Therefore, the non-radiographic risk factors identified in this study may be used to screen for concerning cases.

We note that our interpretations may be limited given all our patients were treated with surgery and we did not have a non-surgical cohort for comparison. Also, regular follow-up of these benign lipomatous tumors was highly variable during the study period. In a number of cases, patients either followed-up with their local physicians or were instructed to return on an as needed basis. Whether or not these patients subsequently developed undetected recurrences or were treated for recurrences at another facility is largely unknown. Given the higher recurrence rates and potential for dedifferentiation in ALTs even after excision, routine surveillance with MRI is now recommended.

Differentiating between ALTs and lipomas may be difficult even after good MR imaging and routine histology. The non-radiographic features associated with the diagnosis of ALTs were location deep in the thigh, size at least of 10 cm, and patient age of 60 years or older. These features may be used to alert clinicians to the higher possibility of an ALT and help clinicians determine appropriate management and surveillance strategies.

## Author Contributions

VL: formulation of project, concept development, analysis of data, and manuscript oversight/editing. JB: data generation and collection, analysis of data, and writing and revisions. LM: data generation and writing. LF: statistical methods and analysis and editing manuscript. W-LW: pathology review and review of all specimens. PL: concept development and manuscript review. BM: concept development and manuscript review. AL: pathology review and review of all specimens. RS: concept development. JM: radiographic assessment and review.

## Conflict of Interest Statement

The authors declare that the research was conducted in the absence of any commercial or financial relationships that could be construed as a potential conflict of interest.
